# Bitendinous Palmaris Longus Associated With Double-Headed Accessory Abductor Digiti Minimi: A Case Report

**DOI:** 10.7759/cureus.90486

**Published:** 2025-08-19

**Authors:** Vinay Sharma, Padamjeet Panchal, Ramesh Babu CS

**Affiliations:** 1 Department of Anatomy, Muzaffarnagar Medical College, Muzaffarnagar, IND; 2 Department of Anatomy, All India Institute of Medical Sciences, Patna, Patna, IND

**Keywords:** accessory abductor digiti minimi, anatomical variation, bitendinous palmaris longus, cadaveric study, double-headed abductor digiti minimi, forearm muscles, guyon’s canal, palmaris longus variation, superficial ulnar artery, ulnar nerve compression

## Abstract

The palmaris longus (PL) is one of the most variable muscles in the human body, frequently exhibiting morphological variants or congenital agenesis. Variations in the abductor digiti minimi and vascular anomalies such as a superficial ulnar artery can also occur, but their coexistence is exceptionally rare.

During the routine dissection of an adult male cadaver, an unusual triad of anatomical variations was observed in the right forearm and hand. A bitendinous PL was present, composed of proximal and distal tendinous segments with a central muscle belly. A double-headed accessory abductor digiti minimi was also identified, with the lateral head arising from the distal tendon of the PL and the medial head from the pisiform, with the lateral head traversing Guyon’s canal over the ulnar nerve and artery before uniting with the medial head. In addition, a superficial ulnar artery was observed, originating from the distal brachial artery at mid-arm level, coursing superficially over the PL tendon. A well-developed palmaris brevis, occurring as several distinct bundles, was also noted. On the contralateral side, a bifid PL tendon was noted without an associated accessory abductor digiti minimi variation. A comprehensive appreciation of its anatomical subtleties holds significance not merely in the academic realm but also as a matter of practical importance in both diagnostic evaluation and therapeutic intervention.

## Introduction

The palmaris longus (PL) is a superficial flexor of the forearm that typically originates from the medial epicondyle via the common flexor tendon and forms a short belly with a long, slender tendon. It courses superficially, passing medial to the flexor carpi radialis tendon, and primarily inserts into the palmar aponeurosis, with some fibers blending into the flexor retinaculum. Functionally, it tenses the palmar aponeurosis and aids wrist flexion [1]. Although the PL is considered vestigial due to its small belly [2] and limited function [3], it remains clinically important because of its high variability and frequent absence among forearm flexors. The congenital agenesis of the PL, occurring unilaterally or bilaterally, is the most commonly observed anatomical variation of the muscle. Its prevalence varies significantly among populations, ranging from 16% to 25% in Caucasians [4] to as low as 4% in Mongoloid populations [5], as reported by Bergman et al. [6]. A recent study reported the absence of the PL muscle in 10% males and 5% females out of 120 individuals examined in each group [7]. The prevalence of PL agenesis has been reported at 17.2%, comprising 9.2% unilateral and 8% bilateral cases. Agenesis of the PL was significantly more common on the left side. Moreover, unilateral absence was more frequently observed in males, whereas bilateral agenesis was more prevalent among females [8]. Das et al. reported that, in addition to the commonly observed structural variations of the PL, a noteworthy morphological variant is its presentation as a digastric or bitendinous muscle in approximately 4% of cases [9]. These anatomical variants are of clinical importance, particularly in surgical procedures involving tendon grafts and in the diagnosis of conditions such as carpal tunnel syndrome, where such variations may be implicated.

The abductor digiti minimi (ADM), an intrinsic hypothenar muscle, arises from the pisiform carpal, pisohamate ligament, and tendinous flexor carpi ulnaris and inserts medially onto the base of the little finger’s proximal phalanx and its extensor hood expansion. The ADM is also known for its high anatomical variability [1]. ADM, involved in little finger abduction, shows notable anatomical variability in 22-35% of cases [10]. Variants of ADM, such as accessory slips (aADM) or atypical origins, may alter local anatomy and contribute to ulnar nerve compression within Guyon’s canal. The altered muscle architecture or the presence of accessory slips can narrow or distort the morphology of the fibro-osseous Guyon’s canal at the wrist. As a result, the ulnar nerve, which traverses this canal, becomes susceptible to compression or entrapment, leading to sensory disturbances such as numbness and tingling in the ring and little fingers, as well as motor weakness of the intrinsic hand muscles supplied by the ulnar nerve [11]. A new classification of aADM was proposed based on its composition and the starting point of the muscle portion: Type I, with a proximal fascial segment and muscle beginning at Guyon’s canal, and Type II, entirely muscular from origin to insertion without a proximal fascial portion. An aADM that was anatomically continuous with the PL was considered a PL variant [12].

A rare vascular anomaly was observed in a male cadaver, characterized by a superficial ulnar artery (SUA) arising high from the brachial artery at the mid-arm level, accompanied by the absence of the PL muscle. It is more susceptible to trauma, which can result in bleeding or ischemia. Owing to its abnormal location, it may be mistaken for a superficial vein on inspection or palpation, leading to diagnostic confusion. This variation can also cause misinterpretation of angiographic findings and complicate surgical procedures such as coronary artery bypass grafting, haemodialysis fistula creation, and forearm flap elevation [13].

The simultaneous coexistence of anatomical variations in both the PL and ADM muscles is a rare occurrence. The SUA is an uncommon vascular variation, with an incidence reported between 0.7% and 9.4%, and its coexistence with PL agenesis is considered particularly rare. The combination of variant bitendinous PL with the presence of an aADM and a SUA represents an exceptionally rare anatomical variation. However, when present, this combination may have significant clinical implications, particularly in the surgical planning and diagnosis of neurovascular compression syndromes of the wrist.

## Case presentation

**Figure 1:**
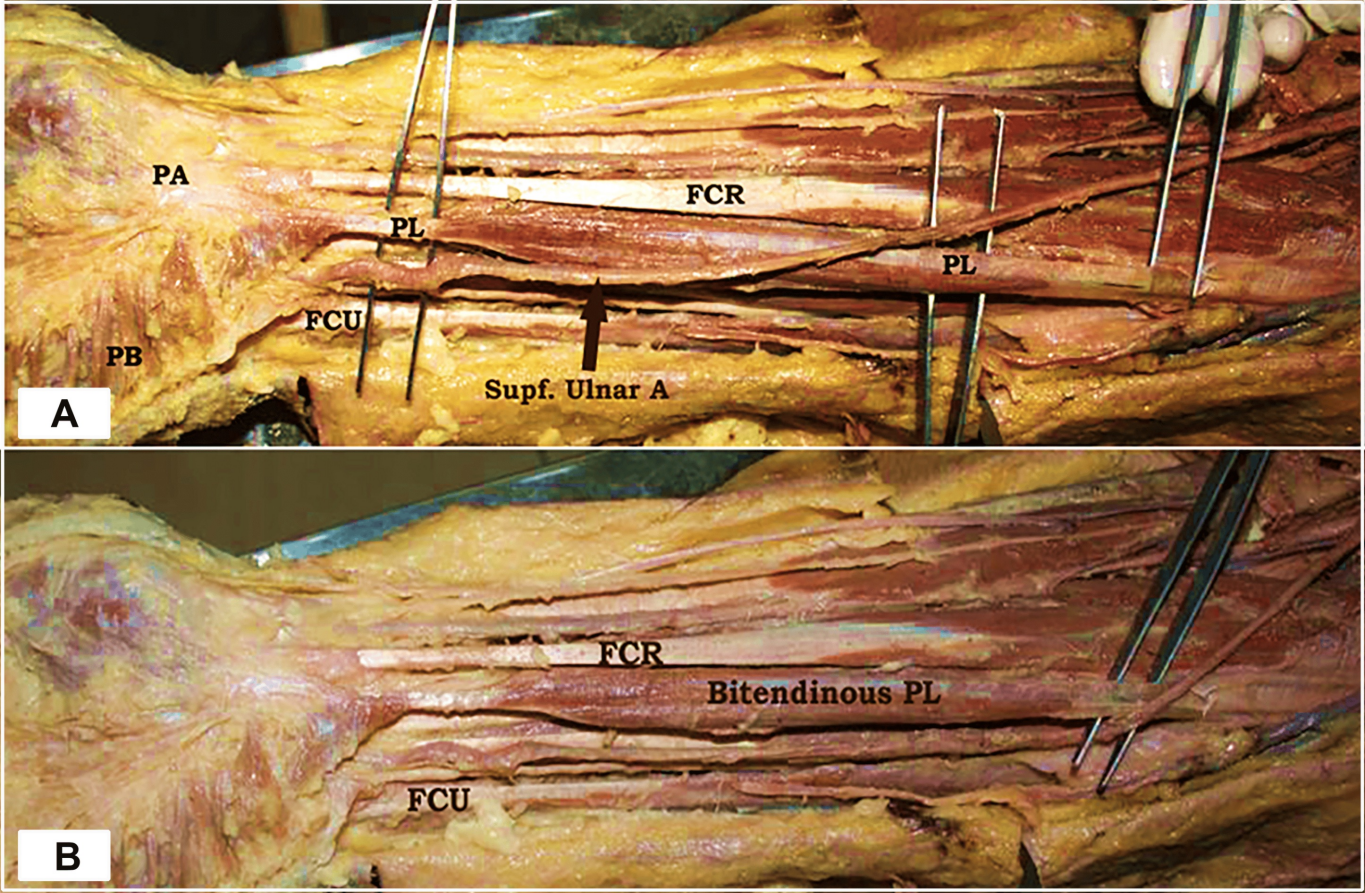
Dissection of the right forearm showing the bitendinous palmaris longus and superficial ulnar artery. (A) Superficial ulnar artery coursing over the tendon of the palmaris longus. (B) The bitendinous palmaris longus with proximal and distal tendinous segments separated by a central muscle belly. PA: palmar aponeurosis; PL: palmaris longus (bitendinous variant); FCR: flexor carpi radialis; FCU: flexor carpi ulnaris; PB: palmaris brevis; Supf. Ulnar A: superficial ulnar artery

During routine dissection classes for undergraduate students, while performing stepwise dissection of a male cadaver, an uncommon anatomical variation was identified on the right side of the forearm and hand, revealing a deviation from the typical muscular or tendinous configuration in this region. A rare bitendinous unilateral variant of the right-sided PL muscle was identified in the forearm. In this variation, the muscle displayed a unique structural configuration, with two distinct tendinous portions: the proximal tendinous component, originating from the medial epicondyle of the humerus; this tendon gave rise to a relatively short muscle belly; and the distal tendinous component, beyond the muscle belly, an elongated tendon descended towards the wrist and merged with the palmar aponeurosis, as in the typical anatomical course (Figure 1).

This dual-tendon arrangement differs from the classic single-tendon morphology of the PL. An uncommon vascular pattern was noted, in which the SUA coursed superficially, passing over the tendon of the PL muscle. This superficial positioning of the artery, rather than its usual deeper course beneath the flexor muscles, represents a rare anatomical variation (Figure 1).

**Figure 2:**
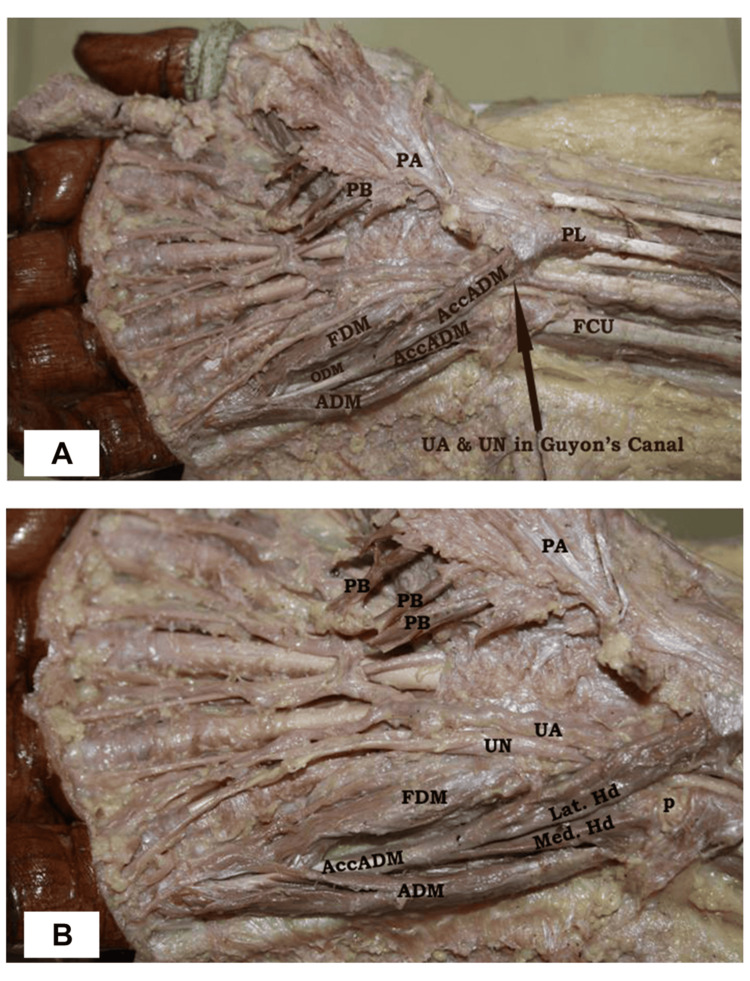
Dissection of the right hand and distal forearm showing accessory abductor digiti minimi and its relation to neurovascular structures in Guyon’s canal. A portion of the palmar aponeurosis, together with the palmaris brevis muscle, has been incised and reflected laterally to expose the underlying hypothenar muscle group. (A) Accessory abductor digiti minimi arising from the distal tendon of the palmaris longus and merging with the main abductor digiti minimi; note the ulnar artery and ulnar nerve within Guyon’s canal. (B) Closer view showing the lateral and medial heads of the accessory abductor digiti minimi and their relationship to the ulnar artery and ulnar nerve. PA: palmar aponeurosis; PB: palmaris brevis; PL: palmaris longus; FCU: flexor carpi ulnaris; AccADM: accessory abductor digiti minimi; FDM: flexor digiti minimi; ODM: opponens digiti minimi; ADM: abductor digiti minimi; UA: ulnar artery; UN: ulnar nerve; Lat. Hd: lateral head; Med. Hd: medial head; P: pisiform

Additionally, an accessory ADM muscle was identified, presenting an unusual double-headed origin. The lateral head of this accessory muscle originated from the distal tendon of the PL, while the medial head arose from the pisiform bone, similar to the normal ADM. The lateral head of the accessory ADM was found to pass through Guyon’s canal, where it crossed both the ulnar nerve and ulnar artery. After emerging from the canal, it joined the medial head distal to the pisiform bone. These two muscular heads then united to form a single common tendon, which coursed distally and was inserted alongside the main ADM into the proximal part of the proximal phalanx of the ulnar finger (Figure 2).

The occurrence of a double-headed accessory ADM is a rare anatomical finding, especially in association with a bitendinous PL and SUA. Interestingly, on the left side of the same cadaver, a bifid tendon of insertion of the PL was observed, without an associated aADM on that side.

## Discussion

Epidemiological analyses from prior investigations have estimated the prevalence of PL tendon agenesis at roughly 15% worldwide, with a demonstrable female predominance and a propensity for symmetrical, bilateral manifestation [14]. The other anatomical variations of the PL includes such as a reversed form with the muscle belly distal and tendon proximal [15], which may produce a wrist bulge, duplication [16], or bifurcation of tendons with separate insertions into structures such as the flexor retinaculum, hypothenar muscles [17], and the presence of an accessory additional PL alongside the normal one [18]. The PL, Type IV arose as a long, thin tendon from the medial epicondyle, transitioned into an elongated muscle belly, and reverted to a tendon above the interstyloid line before inserting into the palmar aponeurosis [19]. Other variations include fascia of the forearm, or carpal bones; aberrant origins from the forearm bones, deep fascia, or adjacent muscles [6]; a digastric form with two bellies connected by an intermediate tendon; partial or complete fusion with neighboring forearm muscles or thenar and hypothenar muscles; and rarer forms such as a hypertrophied PL that may compress nerves, or a triplicate PL with three tendons [6].

Although morphologically and functionally vestigial in humans [3], it nonetheless holds significant surgical and anatomical importance owing to its variability and use in tendon grafting. The bitendinous variant of the PL is distinguished by a centrally positioned fleshy muscle belly interposed between proximal and distal tendinous segments. In their investigation, Rajesh et al. reported this morphological subtype in approximately 2% of examined specimens [20]. A particularly noteworthy case, documented by Lokanathan et al., described a biaponeurotic PL in conjunction with an accessory ADM muscle originating from the distal tendon of the PL on the right side of a male cadaver [21,22]. Another noteworthy morphological variation of the PL is its presentation in a digastric or bitendinous form, reported in approximately 4% of cases [9].

The SUA in this case coursed superficially, crossing over the tendon of the PL muscle. This deviation from its typical deep pathway beneath the flexor musculature represents a rare anatomical variation. Clinically, such a configuration is significant, as a superficially positioned ulnar artery is more susceptible to inadvertent injury during venipuncture, cannulation, or wrist and forearm surgeries. Furthermore, its atypical location may cause diagnostic confusion with superficial veins during inspection or palpation [13].

Dodds et al. reported a 22.4% incidence of anomalous musculature associated with the ulnar canal, encompassing a broad range of aADM configurations. These variants exhibited highly diverse origins, including the pisiform bone, forearm fascia, tendon of the PL, flexor carpi ulnaris, intermuscular fascial septum, fascia covering the flexor carpi radialis, and the modified fascial structure of the flexor retinaculum, thereby highlighting the remarkable anatomical variability of this region [23]. The hypertrophied ADM that can compress the ulnar nerve in Guyon’s canal [24] and fusion with neighbouring muscles hypothenar group of muscles, or ADM along with an aADM originating from the distal part of the PL muscle may contribute to structural crowding within Guyon’s canal and result in neurovascular compression [25,26]. Rixey et al. classified aADM as Type I when fascial proximally with muscle limited to Guyon’s canal, and Type II when muscular throughout its course from origin to canal. The prevalence of an aADM was 24.7%. In 69% of cases, the aADM exhibited fascial morphology proximal to Guyon’s canal, while 31% showed a continuous muscle belly along its entire course. Type II aADM is the variant more likely to produce symptoms due to neurovascular compression [12]. These variations are important in ulnar nerve compression, can mimic soft tissue masses ranging from benign or malignant lesions such as lipomas and hematomas, and may complicate surgical procedures in the hypothenar region [12,27]. Such lesions can be readily identified using ultrasound [28].

The current case presents a unique combination of a bitendinous PL, SUA, and double-headed accessory ADM, with the lateral head arising from the distal PL tendon. This variation may potentially compress structures inside Guyon’s canal, particularly the ulnar nerve, along its branches, predisposing an individual to sensory disturbances, weakness of intrinsic hand muscles, or complicating surgical approaches in this area. Additionally, its association with an anomalous PL tendon suggests a pattern of concurrent muscular variations in the forearm and hand. This rare association highlights the developmental interrelationship between the forearm flexor muscles and the intrinsic muscles of the hand. The superficial course and broad distal insertion of the PL make it well-suited for tendon harvest; however, its considerable anatomical variation necessitates meticulous preoperative evaluation to avoid misidentification and safeguard adjacent neurovascular structures. This complex anatomical variant has significant implications in clinical, surgical, and radiological contexts and contributes to the growing documentation of such rare but important anomalies [9,10].

## Conclusions

This cadaveric study documents a rare triad of variations in the right forearm and hand - a bitendinous PL, a superficially coursing SUL, and a double-headed aADM traversing Guyon’s canal. These anomalies can substantially modify the local anatomy, predispose to neurovascular compression syndromes such as Guyon’s canal syndrome, and complicate procedures including tendon grafting, vascular cannulation, and decompression surgeries. On the contralateral side, a bifid PL tendon was observed without associated ADM variation, highlighting the asymmetrical nature of these findings. Detailed anatomical knowledge and heightened awareness of such variations are crucial for avoiding diagnostic pitfalls and ensuring safe, effective interventions.
